# Associations between Circulating Inflammatory Biomarkers and Indicators of Muscle Health in Older Men and Women

**DOI:** 10.3390/jcm10225316

**Published:** 2021-11-15

**Authors:** Oscar Bergens, Andreas Nilsson, Fawzi Kadi

**Affiliations:** School of Health Sciences, Örebro University, 702 81 Örebro, Sweden; Andreas.nilsson@oru.se (A.N.); Fawzi.kadi@oru.se (F.K.)

**Keywords:** muscle mass, muscle strength, aging, inflammatory biomarkers, obesity, protein intake, physical activity, muscle strengthening activities

## Abstract

Systemic inflammation is believed to contribute to declining muscle health during aging. The present study aims to examine associations between indicators of muscle health and pro- and anti-inflammatory biomarkers in older men and women, while also considering the impacts of physical activity and protein intake. An assessment of skeletal muscle index (SMI) by bioelectrical impedance analysis, handgrip strength, and 5-sit-to-stand time, using standardized procedures, was conducted in a population of older men (*n* = 90) and women (*n* = 148) aged 65–70 years. The inflammatory biomarkers C-reactive protein (CRP), fibrinogen, interleukin (IL)-6, IL-10, IL-18, tumor necrosis factor (TNF)-α, monocyte chemoattractant protein-1, and macrophage inflammatory protein-1α were assessed in blood samples. Data were analyzed and stratified according to biological sex using multiple linear regression models. In older women, SMI was inversely associated with the pro-inflammatory markers CRP (*β* = −0.372; *p* < 0.05), fibrinogen (*β* = −0.376; *p* < 0.05), and IL-6 (*β* = −0.369; *p* < 0.05). Importantly, these associations were independent of abdominal adiposity (waist circumference), protein intake, physical activity level, as well as any adherence to muscle strengthening guidelines (≥2 sessions/week). In contrast, no corresponding associations were observed in men. In conclusion, our findings indicate the detrimental influence of a pro-inflammatory environment on muscle health regardless of important lifestyle-related factors in older women. However, the lack of such associations in older men highlights the importance of considering biological sex when examining the complex interaction between the systemic inflammatory environment and muscle health.

## 1. Introduction

The aging process is associated with a gradual loss of skeletal muscle mass and a concomitant decline in muscle strength and function, which may lead to the development of sarcopenia [1]. Impaired muscle health in older adults has been associated with adverse health outcomes including physical disability, increased risk of falls, fractures, and mortality [2,3]. Several underlying determinants have been proposed to explain age-related decline in muscle health, including hormonal, neural, and behavioral (physical activity and nutrition) factors [4]. Recent research has also suggested that changes in the systemic inflammatory environment may contribute to the deterioration of muscle health in older adults [5]. Slight yet chronic elevations in circulating levels of cytokines, such as interleukin-6 (IL-6), IL-18, tumor necrosis factor (TNF)-α, monocyte chemoattractant protein (MCP)-1, and acute-phase proteins C-reactive protein (CRP) and fibrinogen contribute to a pro-inflammatory environment in older adults [6,7,8,9,10]. The systemic environment in older adults is also influenced by IL-10, which has been shown to play an important anti-inflammatory role by inhibiting the production of pro-inflammatory cytokines by monocytes [11]. While few reports have investigated the associations between IL-10 and muscle health in humans, modest increases in muscle IL-10 mRNA have been reported in healthy older males compared to younger ones [12]. Moreover, elevated levels of IL-10 have been positively associated with the presence of sarcopenia in older men and women [13].

Evidence has shown that there are associations between elevations in IL-6, TNF-α, CRP, and fibrinogen levels and the loss of muscle mass, reduced muscle strength, and poorer overall muscle function [14,15,16,17,18,19,20,21,22]. Population-based evidence indicates that elevated levels of IL-6 and TNF-α contribute to the loss of muscle function via direct effects on muscle catabolism [23]. Interestingly, associations between levels of fibrinogen and physical function appear to be stronger in older women compared to men [17,24], leading to the hypothesis that links between the inflammatory environment and muscle health are sex specific. There is also an interplay between systemic inflammation and metabolic abnormalities such as abdominal obesity, where older adults with increased metabolic risk appear to have higher levels of pro-inflammatory biomarkers [25,26]. Abdominal adiposity is further associated with elevations in levels of CRP, IL-6, IL-18, and macrophage inflammatory protein (MIP)-1α [25]. Moreover, MIP-1α, IL-6, and TNF-α are associated with the progression of obesity-related insulin resistance [7], which has been demonstrated to drive muscle loss by impairing protein synthesis and inducing muscle catabolism through raised muscle cortisol levels in healthy older adults [12,27]. Adiposity-dependent associations between systemic inflammation and physical function have also been demonstrated [16,19,21]. Importantly, mice models with muscle-specific overexpression of IL-10 have been associated with decreased obesity-induced and age-related muscle inflammation and insulin resistance [28,29]. Taken together, the current literature suggests that abdominal obesity should be considered as a factor when examining the impact of the systemic inflammatory environment on muscle health in older adults.

Physical activity (PA), of both an aerobic- and muscle-strengthening nature, and protein intake are commonly identified as two key factors contributing to the prevention of accelerated age-related muscle wasting and physical function [30,31,32,33,34,35,36]. Therefore, these lifestyle-related factors need to be taken into account when elucidating links between muscle health and the systemic inflammatory environment.

The present study aims to investigate associations between indicators of muscle health and systemic inflammation, including pro- and anti-inflammatory biomarkers in older men and women, while also considering the impact of abdominal obesity, protein intake, and PA.

## 2. Materials and Methods

### 2.1. Participants

A total of 252 community-dwelling older men and women (65–70 years old) were recruited from an urban area in Sweden within the framework of the EURODIET project. A description of these participants has already been defined [37]. All clinical investigations were conducted in accordance with the declaration of Helsinki, and written informed consent was obtained from all participants. The study was approved by the regional ethical review board of Uppsala, Sweden.

### 2.2. Assessment of Anthropometry

Height measured to the nearest 0.5 cm and body weight measured to the nearest 0.1 kg were assessed by a portable stadiometer and digital scale, respectively. Waist circumference (WC) was measured to the nearest 0.1 cm at the midpoint between the iliac crest and lower costal margin. Skeletal muscle mass index (SMI) was assessed by bioelectrical impedance analysis (Tanita MC-780 AM, Tanita Corporation, Tokyo, Japan). Skeletal muscle mass was calculated according to the equation provided by Janssen, et al. [38] and expressed in relation to body weight to obtain SMI (kg/BW).

### 2.3. Assessment of Muscle Strength

Handgrip strength of the dominant arm was defined as the best of three attempts using a Jamar handheld dynamometer (Patterson Medical, Warrenville, IL, USA) and was expressed in relation to body weight. A five sit-to-stand (5-STS) test, which involved standing fully upright and sitting down on a chair five times, was performed and was timed and measured in seconds.

### 2.4. Assessment of Protein Intake

Assessment of protein intake was achieved using a validated food frequency questionnaire [39,40]. Daily total protein intake was derived and expressed in relation to body weight.

### 2.5. Assessment of Physical Activity Behaviors

Accelerometer-based assessment of PA was monitored with the Actigraph GT3x activity monitor (Actigraph, Pensacola, FL, USA) over seven days as described previously [37]. Briefly, participants were instructed to wear the monitor around the waist during waking hours. Average accelerometer counts per minute (CPM) were retrieved as a measure of total PA.

Assessment of muscle-strengthening activities (MSA) was achieved with the EPAQ2 questionnaire [41]. Participants were asked to report on the frequency of their engagement in MSA during the last 12 months and were subsequently classified into two groups based on current guidelines on MSA for older adults: less than 2 sessions per week vs. 2 or more sessions per week [42].

### 2.6. Assessment of Laboratory Biomarkers

Blood samples were collected by venipuncture between 8.00 and 10.00 a.m. after an overnight fast. High-sensitivity CRP was measured using fully automated immunoturbidimetric assay. Fibrinogen was determined using an automated immunoassay method with a polyclonal rabbit anti-human antibody (Dako, Glostrup, Denmark). IL-6, IL-10, IL-18, TNF-α, MCP-1, and MIP-1α were analyzed using the Olink Proseek Multiplex Inflammation panel (Olink, Uppsala, Sweden) by qualitative PCR as presented previously [37]. In this assay, matched antibody pairs with complementing DNA oligonucleotide tails were bound to the target protein in the sample. A new PCR target sequence specific to the target protein was formed by a proximity-dependent DNA polymerization event. The resulting sequence was subsequently detected and quantified using standard RT-PCR.

### 2.7. Statistical Analysis

Data are presented as means ± SD. Assessment of normality was achieved through visual inspection of probability plots and the Shapiro–Wilk test of normality. To fit a normal distribution, all variables were transformed when necessary. Differences between sexes were determined by an independent sample t-test and chi-square test. Associations between muscle mass, handgrip strength, 5-STS, and inflammatory biomarkers were investigated using linear regression models. To retrieve comparable effect outcomes from variables with different original units of measurement, all variables were standardized (z-score) before analysis. First, regression models, stratified by sex and adjusted for WC, age, and prescribed medication use, were conducted (Model 1). Second, covariates were added in a stepwise manner according to the following: model 1 + protein intake (Model 2), model 2 + CPM (Model 3), and model 3 + adherence to MSA (Model 4). Assumptions behind linear regression between independent variables, including linearity, homoscedasticity, and multicollinearity, were checked. Level of significance was set to *p* < 0.05. All statistical analyses were performed using SPSS version 27. A priori power calculation revealed a small to moderate effect size, detected with a power of ≥80% given the current sample size and an alpha level of 0.05.

## 3. Results

A total of 238 community-dwelling older adults (148 women and 90 men; men: 67.4 ± 1.5 years; women: 67.4 ± 1.6 years) were included in the final analysis. Six women and eight men had either missing data on all inflammatory biomarkers or body composition, were current smokers, or used prescribed anti-inflammatory medication, and were therefore excluded from the study. Data on anthropometry and indicators of muscle health are presented in Table 1. Sex-specific differences (*p* < 0.05) were observed in all variables except for 5-STS (Table 1). There was no significant difference in average CPM (men: 376.0 ± 111.8 counts; women: 375.0 ± 136.8 counts), adherence to ≥2 sessions per week of MSA (men: 26%; women: 31%), or average protein intake per kg of body weight (men and women: 1.0 ± 0.3 kg/BW) between sexes. Compared to men, women had significantly lower levels of the pro-inflammatory biomarker IL-18 and MIP-1α, as well as lower levels of the anti-inflammatory biomarker IL-10 (all *p* < 0.05) (Table S1). No statistical difference was observed between sexes for the pro-inflammatory biomarkers TNF-α, IL-6, and MCP-1 (Table S1). We have previously reported similar levels of the inflammatory biomarkers CRP and fibrinogen between sexes in this population [37].

We sought to determine the association between indicators of muscle health and levels of inflammatory biomarkers in older adults. In older women, higher SMI was associated with lower levels of the pro-inflammatory biomarkers CRP, fibrinogen, and IL-6 (*p* < 0.05) (Table 2). A longer completion time during 5-STS tests was associated with a higher level of the pro-inflammatory biomarker TNF-α (*p* < 0.05) in older women (Table 2). All significant (*p* < 0.05) observations remained after making adjustments for age, WC, medication use, protein intake, CPM, and MSA (Table S2). No significant associations were observed between muscle health and the inflammatory biomarkers IL-10, IL-18, TNF-α, MCP-1, and MIP-1α in older women (Table 2).

In contrast to the observations made in women, there were no significant associations between indicators of muscle health and biomarkers of systemic inflammation in older men (Table 3).

## 4. Discussion

The present study highlights sex-specific links between indicators of muscle health and biomarkers of systemic inflammation in older adults. A key finding is that SMI was inversely associated with levels of the pro-inflammatory biomarkers CRP, fibrinogen, and IL-6 in older women in accordance with the stated hypothesis. Importantly, abdominal adiposity, protein intake, daily PA, and adherence to MSA guidelines did not offset the inverse associations between SMI and biomarkers of systemic inflammation in women. In contrast, no corresponding associations were observed in older men, denoting the important role of biological sex in the complex interaction between the systemic inflammatory environment and muscle health.

Our results show that associations between systemic biomarkers of inflammation and indicators of muscle health are sex-specific, which strengthens the hypothesis that biological sex plays an important role in the regulation of inflammatory pathways [43]. While the underlying mechanisms behind these sex-specific associations remain to be elucidated, differences in circulating sex hormones have been suggested to be a contributing factor to changes in muscle health and inflammation during aging. For example, it is well established that the male sex hormone testosterone is an anabolic hormone promoting muscle protein syntheses and the maintenance of muscle health [43]. The role of estrogen in muscle health is less conclusive, though it is hypothesized that this sex hormone exerts protective effects on muscle health [44]. Indeed, estrogen has been associated with reduced post-muscle injury inflammation and the promotion of muscle repair processes [44,45,46]. Given the putative protective effects of estrogen on muscle health, it may be hypothesized that the menopause-related sharp reduction in estrogen levels in women, in comparison with less dramatic changes in testosterone levels in men [43], may increase the vulnerability of muscle mass and lead to changes in the inflammatory environment.

An important finding was that additional adjustments by abdominal adiposity, protein intake, CPM, and adherence to MSA did not attenuate observed associations between SMI and systemic inflammation in older women. Generally, adiposity is considered an important factor in inflammation, where greater levels are associated with elevated inflammatory status in older populations [16,26,37,47]. Here, abdominal adiposity was associated with pro-inflammatory biomarkers in older women. However, observed associations between SMI, CRP, fibrinogen, and IL-6 were not attenuated by abdominal adiposity. Our findings are in agreement with previous evidence reporting inverse associations between muscle mass and inflammation with [48,49,50] and without [20,51,52] adjustments for adiposity. Protein intake and PA represent other potent driving factors for muscle wasting and inflammation in older populations [4,30,31,32,33,36,53,54]. Notably, we assessed both the total amount of PA (CPM) and engagement in resistance-type exercises (MSA), as well as protein intake, and this strengthens the evidence that there are independent links between the systemic inflammatory environment and muscle health in older women.

Another important finding was the lack of an association between inflammation and muscle health in older men. The current literature shows conflicting evidence, where some [20,49,50,51,53] but not all studies have reported an association between muscle mass and inflammation in older men [21,48,55]. A recent meta-analysis concluded that muscle loss is more strongly associated with higher levels of CRP, IL-6, and TNF-α in older men compared to women [52]. However, this association is strongly influenced by the presence of age-related diseases (e.g., cardiovascular disease). For example, associations between IL-6 and muscle loss were significantly stronger in populations with cardiovascular disease compared to their disease-free peers [52]. Indeed, previous studies reporting associations between muscle mass and inflammation have included adults with mobility limitations [51], with age-related diseases, and with greater levels of obesity [53] or sarcopenic risk [49,50]. Taken together, given that the health status of the studied population is likely to influence any observed links between systemic inflammation and muscle health, our sample of older men without manifest diseases may partly explain the lack of any associations in the present study.

In contrast to previous data [14,16,20,21,24,49,55,56,57,58], we found only a few links between indicators of muscle function and biomarkers of systemic inflammation. Notably, compared to our sample, participants in previous studies included populations with a higher prevalence of obesity [14,16,56,58], the presence of multiple chronic conditions [14], or sarcopenia [49,55]. Furthermore, in our sample of older adults, very few had a handgrip strength value below the EWGSOP2 cut-offs of <27 kg (men) and <16 kg (women) [1] (men, *n* = 0; women, *n* = 3), indicating an adequate overall physical function. Moreover, some other studies have not made proper adjustments for adiposity level when exploring links between muscle health and systemic inflammation [16,55,57]. Taking this into account, and based on a sample of older adults without manifest diseases and with adequate muscle function, there seem to be only weak links between muscle function and the systemic inflammatory environment after adjustment for adiposity level.

Our findings are strengthened by the consideration of important covariates with the potential to readily impact muscle health. This includes an objectively assessed amount of PA, engagement in specific types of exercise activities (MSA), and protein intake. However, the study is not without its limitations: (1) the cross-sectional design precludes causality, (2) our older men and women are not representative of broader populations of older adults with various health statuses, and (3) the systemic inflammatory environment in older adults is likely to be influenced by a wider set of inflammatory agents that were not covered in this study.

## 5. Conclusions

In conclusion, our findings highlight that biological sex is an important factor to consider when examining the complex associations between biomarkers of systemic inflammation and muscle health in older adults. Importantly, the detrimental associations between pro-inflammatory biomarkers and muscle health in older women were evident regardless of abdominal adiposity, protein intake, PA level, and engagement in MSA, highlighting the role of systemic inflammation as an independent risk factor for the deterioration of muscle health. These findings warrant further investigations to elucidate the molecular mechanisms underlying the sex-specific links observed in this study.

## Figures and Tables

**Table 1:** Anthropometrics and indicators of muscle health.

	Women (*n* = 148)	Men (*n* = 90)
Height (cm)	164.4 ± 5.6	178.8 ± 6.6 *
Weight (kg)	64.5 ± 10.0	81.2 ± 5.6 *
Body mass index (kg/m^−2^)	23.9 ± 3.4	25.4 ± 3.1 *
Waist circumference (cm)	79.9 ± 9.2	94.4 ± 10.1 *
Skeletal muscle index (% BW)	26.6 ± 3.4	34.5 ± 3.3 *
Handgrip (kg/BW)	27.7 ± 5.2	43.7 ± 7.2 *
5-sit-to-stand (sec)	10.4 ± 2.5	10.2 ± 2.1

* *p* < 0.05 vs. women.

**Table 2:** Associations (β-coefficients and 95% CI) between muscle health and pro- and anti-inflammatory biomarkers in older women.

	Skeletal Muscle Index ^a^	Handgrip Strength	5-Sit-to-Stand
CRP ^b^	−0.372 (−0.612 to −0.133) *	−0.130 (−0.302 to 0.043)	−0.054 (−0.219 to 0.112)
Fibrinogen	−0.376 (−0.622 to −0.131) *	−0.150 (−0.327 to 0.026)	−0.144 (−0.312 to 0.023)
TNF-α ^a^	−0.160 (−0.425 to 0.105)	0.103 (−0.082 to 0.287)	0.187 (0.013 to 0.360) *
IL-6	−0.369 (−0.621 to −0.117) *	−0.143 (−0.323 to 0.036)	0.112 (−0.057 to 0.280)
IL-10	−0.017 (−0.288 to 0.254)	0.160 (−0.026 to 0.347)	0.093 (−0.083 to 0.268)
IL-18	−0.123 (−0.385 to 0.139)	0.003 (−0.180 to 0.185)	0.089 (−0.081 to 0.259)
MCP-1	0.112 (−0.157 to 0.382)	−0.058 (−0.246 to 0.130)	0.083 (−0.093 to 0.258)
MIP-1α	0.032 (−0.234 to 0.298)	0.038 (−0.148 to 0.224)	0.158 (−0.015 to 0.330)

^a^*n* = 147. ^b^ *n* = 146. Data adjusted by WC, age, and medication use (Yes/No). * *p* < 0.05. CRP = C-reactive protein. TNF-α = tumor necrosis factor alpha. IL-6 = interleukin-6. MCP-1 = monocyte chemoattractant protein-1. MIP-1α = macrophage inflammatory protein-1α.

**Table 3:** Associations (β-coefficients and 95% CI) between muscle health and pro- and anti-inflammatory biomarkers in older men.

	Skeletal Muscle Index	Handgrip Strength	5-Sit-to-Stand
CRP ^a^	−0.126 (−0.458 to 0.206)	0.025 (−0.242 to 0.292)	−0.051 (−0.267 to 0.165)
Fibrinogen ^a^	−0.225 (−0.555 to 0.106)	−0.041 (−0.226 to 0.309)	−0.139 (−0.354 to 0.076)
TNF-α	−0.134 (−0.477 to 0.209)	−0.139 (−0.414 to 0.137)	−0.092 (−0.316 to 0.131)
IL-6	−0.012 (−0.361 to 0.337)	0.050 (−0.231 to 0.331)	−0.146 (−0.371 to 0.079)
IL-10	0.047 (−0.298 to 0.392)	−0.099 (−0.376 to 0.178)	−0.011 (−0.236 to 0.214)
IL-18	0.326 (−0.016 to 0.667)	0.049 (−0.232 to 0.329)	−0.054 (−0.281 to 0.173)
MCP-1	−0.273 (−0.617 to 0.071)	−0.195 (−0.473 to 0.083)	−0.014 (−0.241 to 0.213)
MIP-1α	−0.046 (−0.394 to 0.302)	−0.109 (−0.389 to 0.170)	−0.004 (−0.223 to 0.231)

^a^*n* = 89. Data adjusted by WC, age, and medication use (Yes/No).

## Data Availability

Data supporting reported results are available upon reasonable request due to ethical principles.

## Supplementary Materials

The following are available online at https://www.mdpi.com/article/10.3390/jcm10225316/s1: Table S1. Biomarkers of systemic inflammation in men and women, and Table S2. Associations (*β*-coefficients and 95% CI) between muscle health and pro- and anti-inflammatory biomarkers in older women.
