# Supplementary material for: Associations between Circulating Inflammatory Biomarkers and Indicators of Muscle Health in Older Men and Women
Source: J Clin Med. 2021 Nov 15;10(22):5316. doi: 10.3390/jcm10225316 (PMC8623342; doi:10.3390/jcm10225316)
Supplement: Supplementary file 1 [file jcm-10-05316-s001.zip › jcm-1446644-supplementary.pdf]

Table S1. Biomarkers of systemic inflammation in men and women.

	**Women (n = 148)**	**Men (n = 90)**
TNF-α^{a} (au)	3.1 ± 0.4	3.2 ± 0.4
IL-6 (au)	3.3 ± 0.6	3.4 ± 0.8
IL-10 (au)	3.7 ± 0.4	3.9 ± 0.4*
IL-18 (au)	8.0 ± 0.5	8.3 ± 0.5*
MCP-1 (au)	12.5 ± 0.4	12.5 ± 0.4
MIP-1α (au)	5.3 ± 0.5	5.5 ± 0.5*

[a]Women n = 147. Au = arbitrary units. *$p < 0.05$ vs. women.

Table S2. Associations (β-coefficients and 95% CI) between muscle health and pro- and anti-inflammatory biomarkers in older women.

	**Skeletal muscle index[a]**	**5-sit-to-stand**
**Model 2**		
CRP[b]	-0.379 (-0.619 to -0.139)*	-
Fibrinogen	-0.377 (-0.625 to -0.130)*	-
TNF-α^{a}	-	0.189 (0.015 to 0.364)*
IL-6	-0.369 (-0.622 to -0.130)	-
**Model 3**		
CRP[b]	-0.330 (-0.590 to -0.070)*	-
Fibrinogen	-0.358 (-0.625 to -0.091)*	-
TNF-α^{a}	-	0.184 (0.003 to 0.364)*
IL-6	-0.375 (-0.648 to -0.102)*	-
**Model 4**		
CRP[b]	-0.311 (-0.582 to -0.040)*	-
Fibrinogen	-0.415 (-0.692 to -0.139)*	-
TNF-α^{a}	-	0.188 (0.007 to 0.369)*
IL-6	-0.364 (-0.648 to -0.040)*	-

[a]n = 147. [b]n = 146. Model 2 = Data adjusted by WC, age, medication use (Yes/No) and protein intake. Model 3 = Model 2 + average counts per minute. Model 4 = Model 3 + Participation in ≥2 muscle strengthening activities per week (Yes/No). *$p < 0.05$. CRP = C-reactive protein. TNF-α = tumor necrosis factor alpha. IL-6 = interleukin-6.
